# Effects of Increased Flight on the Energetics and Life History of the Butterfly *Speyeria mormonia*

**DOI:** 10.1371/journal.pone.0140104

**Published:** 2015-10-28

**Authors:** Kristjan Niitepõld, Carol L. Boggs

**Affiliations:** 1 Department of Biology, Stanford University, Stanford, California, United States of America; 2 Rocky Mountain Biological Laboratory, Crested Butte, Colorado, United States of America; 3 Department of Biological Sciences, University of South Carolina, Columbia, South Carolina, United States of America; Oxford Brookes University, UNITED KINGDOM

## Abstract

Movement uses resources that may otherwise be allocated to somatic maintenance or reproduction. How does increased energy expenditure affect resource allocation? Using the butterfly *Speyeria mormonia*, we tested whether experimentally increased flight affects fecundity, lifespan or flight capacity. We measured body mass (storage), resting metabolic rate and lifespan (repair and maintenance), flight metabolic rate (flight capacity), egg number and composition (reproduction), and food intake across the adult lifespan. The flight treatment did not affect body mass or lifespan. Food intake increased sufficiently to offset the increased energy expenditure. Total egg number did not change, but flown females had higher early-life fecundity and higher egg dry mass than control females. Egg dry mass decreased with age in both treatments. Egg protein, triglyceride or glycogen content did not change with flight or age, but some components tracked egg dry mass. Flight elevated resting metabolic rate, indicating increased maintenance costs. Flight metabolism decreased with age, with a steeper slope for flown females. This may reflect accelerated metabolic senescence from detrimental effects of flight. These effects of a drawdown of nutrients via flight contrast with studies restricting adult nutrient input. There, fecundity was reduced, but flight capacity and lifespan were unchanged. The current study showed that when food resources were abundant, wing-monomorphic butterflies living in a continuous meadow landscape resisted flight-induced stress, exhibiting no evidence of a flight-fecundity or flight-longevity trade-off. Instead, flight changed the dynamics of energy use and reproduction as butterflies adopted a faster lifestyle in early life. High investment in early reproduction may have positive fitness effects in the wild, as long as food is available. Our results help to predict the effect of stressful conditions on the life history of insects living in a changing world.

## Introduction

Many ecological situations increase the need for movement, for example when available resources are few or they are scattered across the landscape. Habitat loss, deterioration and fragmentation can affect the costs and benefits of movement by altering the accessibility of resources and the size, configuration and composition of the individuals’ resource pool. How does increased expenditure on movement affect the life history of individuals? Exercise has many positive effects [1–3], but increased activity can have negative consequences, such as increased oxidative damage or decreased lifespan [4–6]. In this context, animal flight is a particularly interesting trait as the energetic demand of flapping flight is extremely high [7, 8].

In addition, the energy consumed by movement could be allocated to other fitness-related processes: maintenance, reproduction and storage. Resource allocation among these processes is a fundamental part of life history theory. The Y model of allocation [9] has been useful in developing an understanding of allocation trade-offs between different traits. The Y model is a simple model, while in many organisms resource allocation is a complex and dynamic process. First, there are more than two allocation targets, including investment in foraging that enlarges the available resource pool. Second, the total resource pool consists of diverse nutrients that are required at different life-history stages [10].

Resource allocation can be seen as a network with pushing and pulling forces. Food intake pushes resources into the pool from which various processes pull energy and nutrients. Restricting the size of the resource pool can have dramatic effects on allocation patterns. For example, chronic caloric restriction reduces fecundity in many animals while lifespan is prolonged [11]. In our study organism, the butterfly *Speyeria mormonia* (Nymphalidae), lifespan is not affected by adult food restriction but reproductive output depends on adult feeding [12, 13]. Here we examine the effects of a pulling force on the network, through increased flight. Studies on wing dimorphic species such as crickets have demonstrated that there can be strong trade-offs between flight and reproduction, leading to the so called flight-oogenesis syndrome [14, 15]. However, it is unclear how prominent these trade-offs are in wing-monomorphic taxa such as flies and butterflies that fly throughout their adult lives [16, 17].

Here, we examined the effect of repeated, forced flight on the life history of *Speyeria mormonia*. We kept females in individual cages and throughout their lives recorded reproductive output, egg dry mass and composition, body mass, resting metabolic rate, flight metabolic rate, food intake, wing area, and lifespan. We predicted that increased flight would either lead to a similar reduction in fecundity as that observed when experimentally reducing the sugar water intake of adult females, or result in compensatory feeding, or some combination of the two.

## Materials and Methods

### Ethics statement

This work was done under USDA APHIS Permit #P526P-12-03928 for interstate transport of Lepidoptera including *Speyeria mormonia*, and CDFA No. 43-07-12 for importation of Lepidoptera into California. Permissions to work on private land for the field component of this study are on file at the Rocky Mountain Biological Laboratory. No other permits were required.

### Rearing

*Speyeria mormonia* is a montane butterfly with a broad distribution across western North America. We collected wild females in the vicinity of the Rocky Mountain Biological Laboratory, Crested Butte, Colorado (38°57’N, 106°58’W, 2900 m asl) and reared their offspring in sibling groups in the greenhouse with abundant food under standard conditions [13] (S1 Appendix: Detailed Methods). Adult females were mated with unrelated males in the afternoon of the day of emergence. Females were kept in cylindrical glass cages lined with wax paper (height 20 cm, diameter 15 cm). The cages contained moist paper towel and one host plant leaf inserted in a small water-filled vial. The species does not generally lay eggs directly on the host plant leaves and in our cages eggs could be found on all surfaces.

### Flight treatments

A total of 40 females from 14 families were used in the experiment. We assigned 20 adult females to the flight treatment and 20 to the control. We aimed to allocate size-matched siblings to both treatments, but only one female was available in five families. Each morning, up to four females at a time were placed in a 30 x 30 x 30 cm cage and stimulated to fly by gently sweeping them with a fine paintbrush. The temperature in the cage was 28°C during the trials. The cage walls were lined with plastic film, which eliminated physical damage to the butterflies and reduced their tendency to cling to the cage walls. Each butterfly was flown daily for 3 x 4 min with 5 min of rest in between flight bouts. During the flight period, we kept each individual flying as continuously as possible by sweeping it with the paintbrush when it landed. During the rest period, we shaded the flight cage with black plastic to prevent spontaneous flight.

We chose the repeated 4 min flight treatments instead of longer continuous flight because we were interested in the effect of energy consumption during flight, not effects of exhaustion and fatigue. Even though 12 min of additional flight might seem trivial, at a typical flight speed of 2.5–4 m/sec the butterfly would travel 1800–2880 m in that time in windless conditions. *Speyeria mormonia*, as well as many other medium-sized temperate butterflies typically fly in short bursts. One of us (CLB) performed flight activity observations of wild *S*. *mormonia* females in 1985 and 1986. The observations were distributed as evenly as possible between 9:00–17:00. Captured and cooled females were re-released in the field and the activity of each individual was recorded starting from the first time the butterfly moved. In 1985, 61 females were observed for a total of 11.47 h, and in 1986 26 females were followed for 9.21 h. The time spent flying increased with female age (estimated from wing wear) and ranged from 2 to 3.5% of the observation period in 1985 and 2.5 to 5% in 1986. Combined across both years, the observational time consisted of ~60% full sunlight, ~16% partial sun, and 24% cloudy/shade. A rough extrapolation over eight hours of available flight time gives an estimate of a total of 10 to 17 min of flight per female per day in 1985 and 12 to 24 min in 1986. Taken together, these observations suggest that our flight treatment did represent a large fraction of the daily flight time of a *S*. *mormonia* female, possibly even up to a full day’s flight activity.

### Respirometry

We measured resting and flight metabolic rate as CO_2_ emission rate using flow-through respirometry every third day throughout the life of all individuals (see S1 Appendix: Detailed Methods and Niitepõld et al. [13] for details). We calculated the peak flight metabolic rate (PMR), which is the highest CO_2_ production rate, and the flight metabolic rate (FMR), which is the total volume of CO_2_ emitted during the flight trial and which reflects overall flight performance, including endurance.

### Feeding and measuring food intake

We fed all females twice a day on a 1:3 v:v sugar-water solution. Butterflies were fed after the flight treatment in the morning in haphazard order. We held the butterfly by its wings with forceps and provided a droplet of sugar-water on a plastic plate in front of the butterfly. We extended its proboscis into the droplet using a needle and once it was feeding, we removed the forceps and allowed the butterfly to feed *ad libitum*. The afternoon feeding took place after the measurements of metabolic rate. Food intake was measured every third day in individuals subjected to respirometry trials that day. We weighed the individual prior to and after feeding using a high-precision 0.1 mg scale (Sartorius AG, Göttingen, Germany) and calculated the mass of the consumed sugar water.

We compared measured sugar water intake to predicted intake. To predict the sugar water intake of females in the flight treatment, we modelled the intake of their siblings in the control treatment as a function of age and extracted least squares means of sugar water consumption for each family. To this baseline value we added the amount of sugar water corresponding to the amount of energy used in flight, which was calculated based on the respirometry trials (see S1 Appendix: Detailed Methods for details).

### Fecundity and lifespan

Every afternoon we collected and counted eggs laid by individual females. The eggs were dried at 50°C for 72 hours. We collected dead females every morning and afternoon.

### Egg chemistry

We quantified the lipid, glycogen and protein content, and dry mass of eggs collected at ages 4, 5, 7, 11, 13, 17, and 19 days. Egg protein content was quantified for one egg from each age using the Bradford dye-binding method following modified protocols of Giron et al. [18] (see S1 Appendix: Detailed Methods). We quantified the glycogen content of a pooled sample of 4 eggs from each age following a modified protocol of Giron et al. [18] (see S1 Appendix: Detailed Methods). For egg triglycerides we used a pooled sample of 3 to 4 eggs from each age using the standard protocol for Infinity Triglycerides Stable Reagent (ThermoScientific, Waltham, MA, USA) (see S1 Appendix: Detailed Methods). All results were expressed as μg per egg.

### Statistical analyses

We modelled longitudinal data (body mass, RMR, PMR, FMR, food intake, daily egg production, egg dry mass, wing area) with mixed general linear models using the ‘repeated’ statement in Procedure Mixed in SAS 9.2 (SAS Institute, Cary, NC, USA). We used autoregressive(1) or heterogeneous autoregressive(1) as the covariance structure, depending on which one resulted in the lowest Akaike Information Criterion (AIC) value. Egg chemistry results were analysed using Procedure Glimmix and the sp(pow) covariance structure to account for unequal sampling intervals. We used backward elimination to drop non-significant terms from the initial full model. We omitted data from day 22 onward because of declining sample size at older ages. Lifespan and total number of eggs were modelled using ANCOVA with body mass as a covariate and family and treatment as factors.

## Results

### Body mass

**Fig 1:**
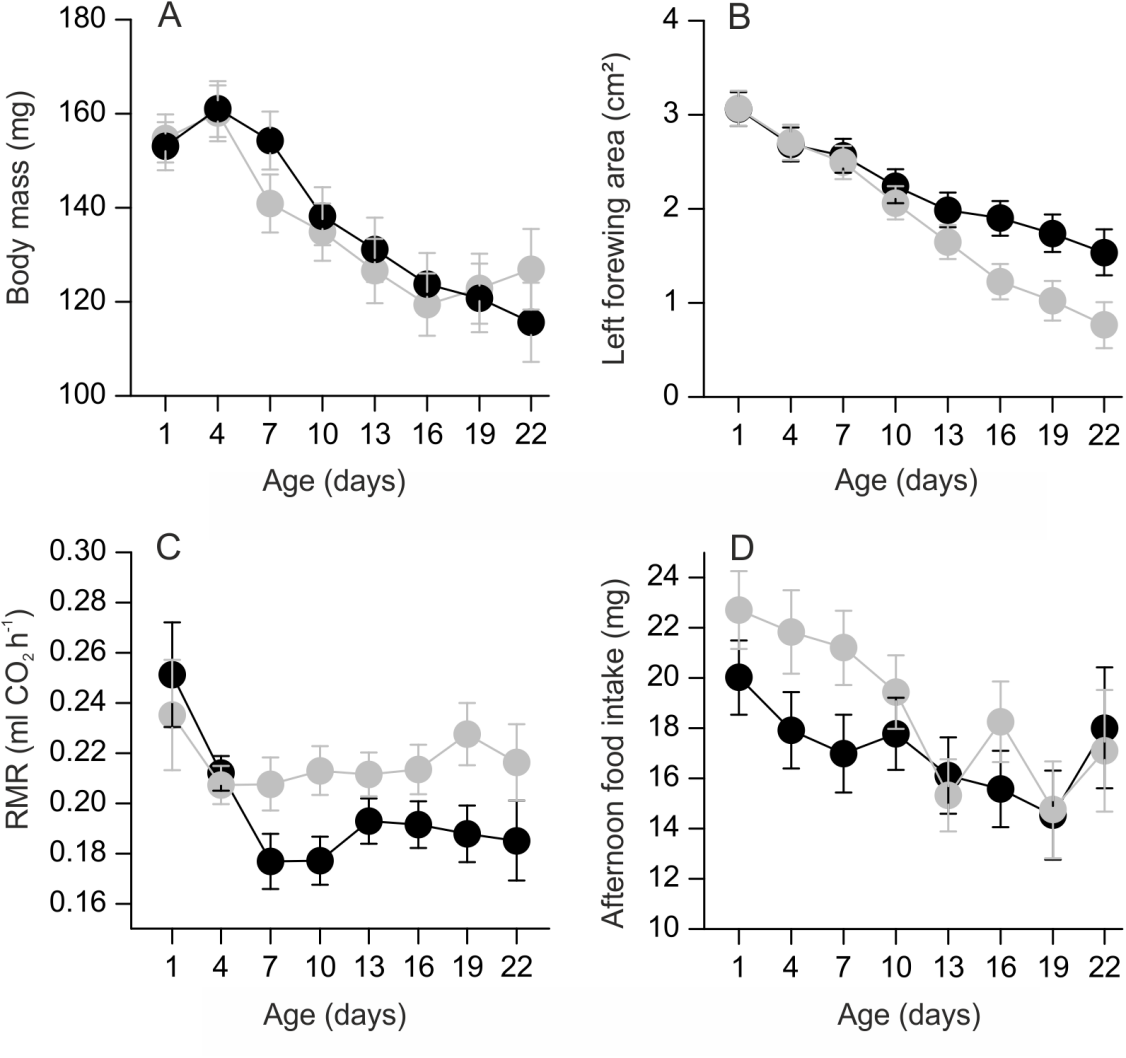
Effect of flight on body mass, wing area, resting metabolic rate and food intake. a) Body mass decreased with age, but did not differ between females in the flight treatment (grey circles) and control females (black circles). b) Females in the flight treatment tended to experience faster wing wear than control females. C) The flight treatment elevated resting metabolic rate (RMR). The data are size-adjusted least squares means from a repeated measures mixed model. The mean measurement temperature was 31.6°C (s.d. 0.4). d) Sugar water intake was higher among females in the flight treatment.

Wet body mass decreased with age (*F*_1,216_ = 62.23; *p* < 0.0001) (Fig 1A). The flight treatment had no significant effect on body mass (*F*_1,24_ = 0.59; *p* = 0.51) and the interaction between age and treatment was not significant (*F*_1,216_ = 0.05; *p* = 0.83). There was a significant effect of family on body mass (*F*_13,24_ = 4.57; *p* = 0.006) (S1 Fig).

### Wing area

Wing area decreased with age (*F*_1,215_ = 113.17; *p* < 0.0001). The treatment main effect was not significant (*F*_1,38_ = 0.16; *p* = 0.69), but there was a near-significant treatment by age interaction effect (*F*_1,215_ = 3.51; *p* = 0.06). The interaction suggested more rapid wing degradation in females that were forced to fly every day compared to control females (Fig 1B). The effect of family on wing area was non-significant (*F*_13,24_ = 1.08; *p* = 0.42).

### Resting metabolic rate

Body mass and measurement temperature had a positive effect on RMR (Table 1). Individuals had lower RMR as they grew older (Fig 1C). A significant age by treatment interaction indicated that females that were forced to fly had generally higher RMR than controls, particularly as they aged. RMR decreased as time since the previous sugar-water feeding increased. There was a significant effect of family on RMR (S2 Fig).

**Table 1:** Factors affecting resting metabolic rate.

Independent variable	DF	*F*	*P*
Family	13,24	3.58	**0.003**
Body mass	1,207	47.16	**<0.0001**
Age	1,207	2.12	0.15
Temperature	1,207	19.18	**<0.0001**
Flight treatment	1,24	0.04	0.85
Time since feeding	1,207	5.04	**0.3**
Age x treatment	1,207	4.65	**0.03**

RMR was modelled using a repeated measures mixed model with heterogeneous autoregressive (1) covariance structure.

### Peak and flight metabolic rate

**Fig 2:**
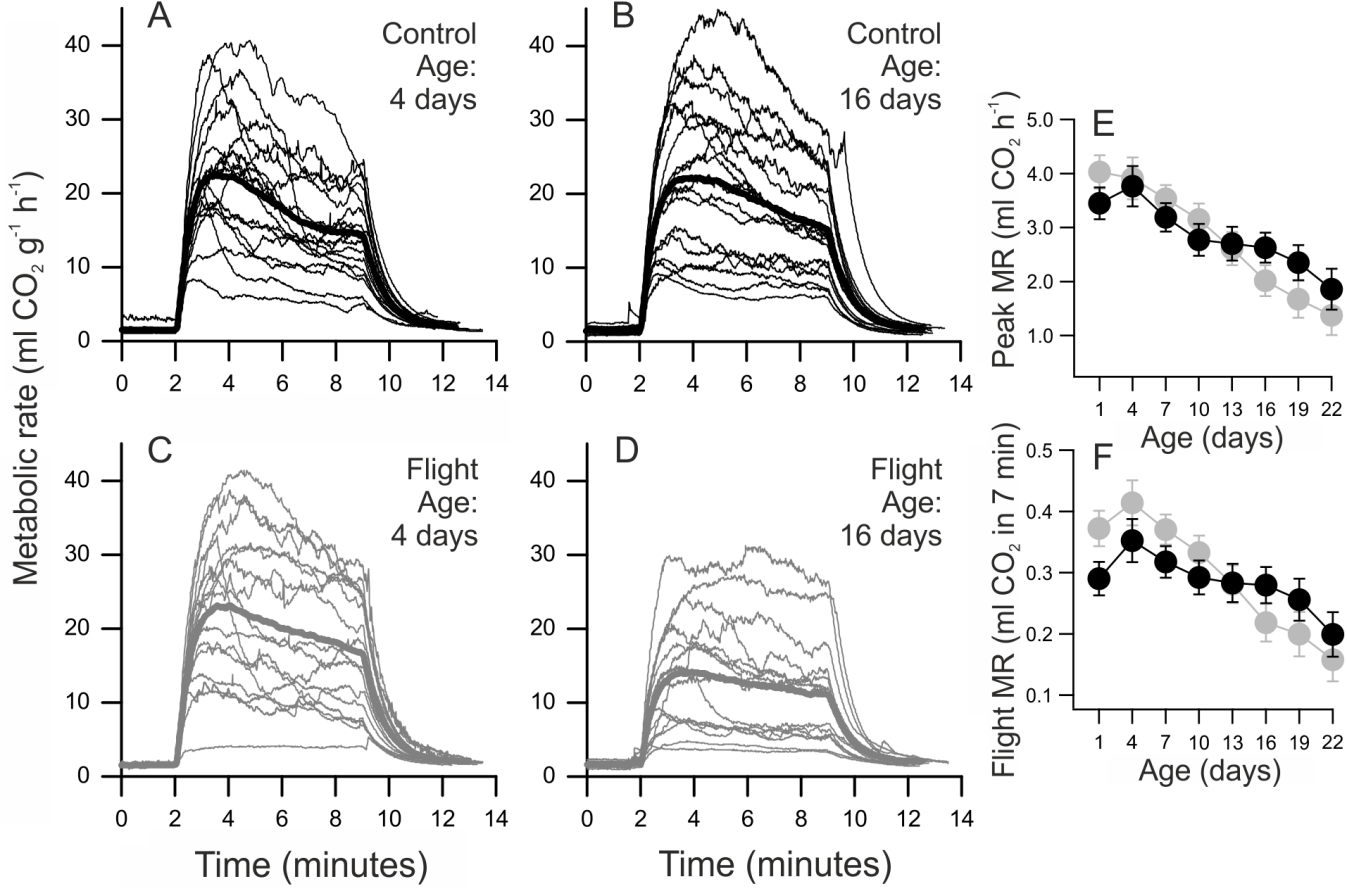
Effect of flight treatments on flight metabolic rate. a-d) CO_2_ emission rate plotted against time at ages 4 and 16 days in the control (upper panels) and flight treatment (lower panels). For illustrative purposes, metabolic rate has been divided by body mass. The thick line in each panel represents mean CO_2_ emission rate. The first 1.5 min of the recording represented RMR. Peak flight metabolic rate (PMR) is typically reached during the first minutes of the flight measurement. The mean measurement temperature was 31.7°C (s.d. 0.4). Panel e) depicts the least squares means of PMR in control females (black circles) and flight treatment females (grey circles) plotted against age. Panel f) shows the total volume of CO_2_ emitted during the 7 min flight measurement. In both cases, the interaction between treatment and age is significant.

Peak metabolic rate increased with body mass, and decreased with age (Table 2) (Fig 2A–2D). A significant age by treatment interaction indicated that individuals that were forced to fly experienced a stronger decline in PMR than did controls (Fig 2E). There was a significant family effect on PMR (S3 Fig). Our initial model contained wing area as a covariate but the effect was not significant (*F*_1,212_ = 2.05; *p* = 0.15) and it was therefore dropped from the final model.

**Table 2:** Factors affecting peak metabolic rate (PMR) and flight metabolic rate (FMR).

	A) PMR			B) FMR		
Independent variable	DF	*F*	*P*	DF	*F*	*P*
Family	13,24	3.59	**0.003**	13,24	3.41	**0.005**
Body mass	1,215	22.76	**<0.0001**	1,215	19.68	**<0.0001**
Age	1,215	45.70	**<0.0001**	1,215	12.03	**0.0006**
Flight treatment	1,24	0.13	0.72	1,24	0.40	0.53
Age x treatment	1,215	6.84	**0.01**	1,215	5.46	**0.02**

PMR and FMR were modelled using a repeated measures mixed model with heterogeneous autoregressive (1) covariance structure.

Flight metabolic rate increased with body mass and decreased with age (Table 2B). FMR decreased with age more strongly in the flight treatment compared to the control (Fig 2F). We detected a significant effect of family on FMR. Wing area had no significant effect on FMR (*F*_1,212_ = 1.41; *p* = 0.24) and was not included in the final model.

### Food intake

Sugar-water intake decreased with age (*F*_1,206_ = 21.68; *p* < 0.0001). Intake increased with time since the previous feeding event (*F*_1,206_ = 25.30; *p* < 0.0001). Females that were forced to fly had higher food intake than controls (*F*_1,24_ = 5.76; *p* = 0.02; Fig 1D). The age by treatment interaction was not significant (*F*_1,206_ = 1.87; *p* = 0.17). Family had a significant effect on food intake (*F*_13,24_ = 3.48; *p* = 0.004). Fig 1D suggests that there was already a difference in food intake at age 1 when individuals in the flight treatment had not yet been flown. However, the difference between the two groups was not significant on day 1 (*F*_1,36_ = 2.27; *p* = 0.14), whereas the difference was significant on days 4 and 7 (*p* = 0.02 on both days).

We calculated the estimated energy consumption during forced flight for each individual for each day and compared the predicted sugar water intake to the measured afternoon intake. The mean afternoon sugar water intake was 16.8 ± s.d. 5.1 mg in the control group. The predicted mean intake for females in the flight group based on extra flight was 19.4 ± 4.8 mg whereas the measured arithmetic mean was 20.4 ± 6.9 mg. The predicted intake was thus 15.6% higher than the intake of controls, but the measured intake was 21.4% higher than the intake of control females.

### Lifespan

The flight treatment had no significant effect on lifespan (*F*_1,23_ = 0.15; *p* = 0.70). The median lifespan was 21 days in both groups. The mean was 22.2 ± s.d. 6.6 days among control females and 19.9 ± 4.3 days among flight treatment females. Neither family (F_13,23_ = 1.08; *p* = 0.42) nor initial body mass (F_1,23_ = 0.35; *p* = 0.56) significantly explained variation in lifespan.

### Fecundity

**Fig 3:**
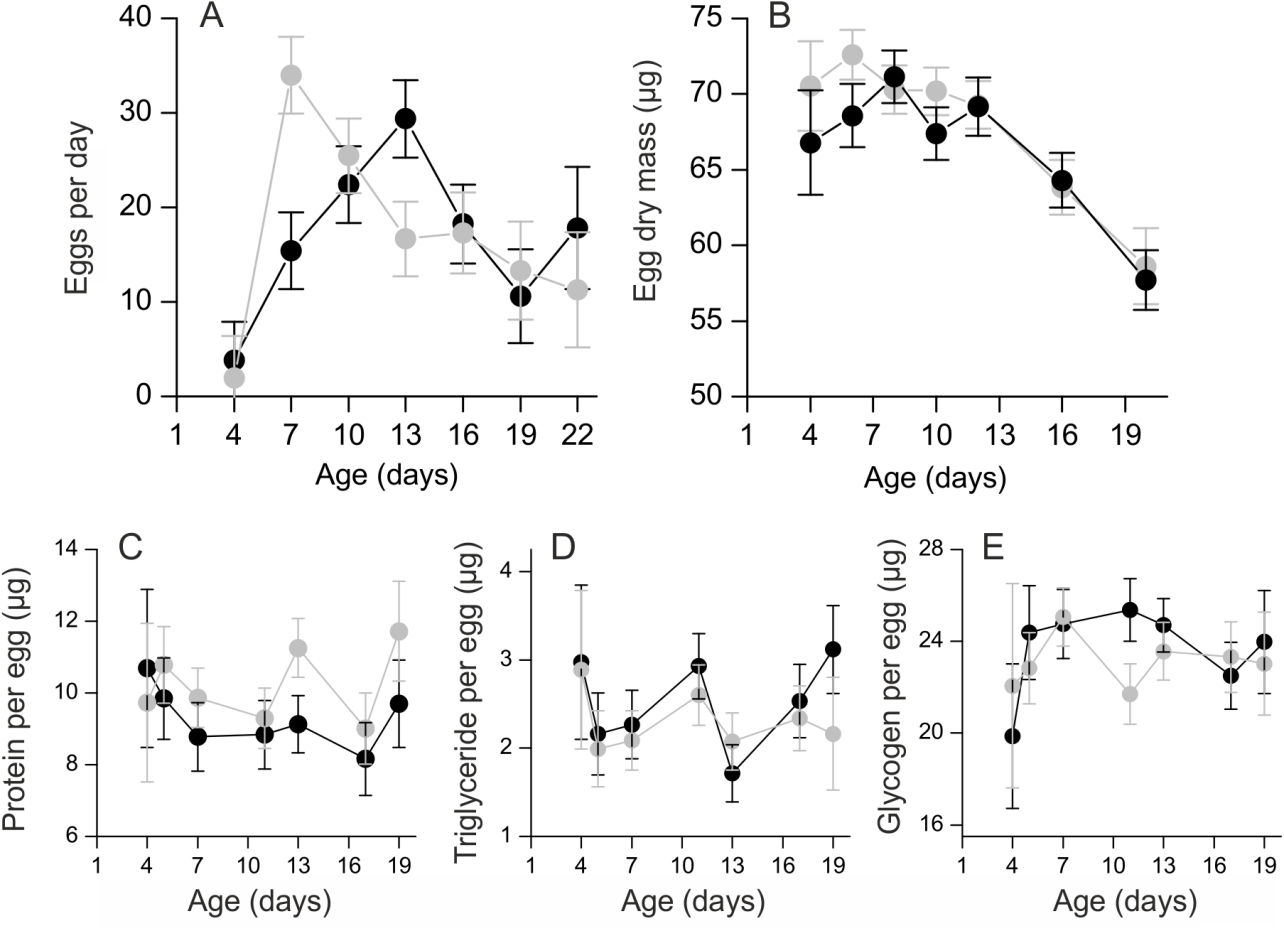
Effect of flight on egg number, mass and composition. a) The number of eggs laid followed a nonlinear pattern where daily egg production first increased, then decreased. Females in the flight treatment (grey circles) had higher early-life fecundity than control females (black circles). b) Egg dry mass decreased with female age but was not affected by the flight treatment. Flight also had no effect on the c) protein content, d) triglyceride content, or e) glycogen content of eggs. The data are least squares means ± standard error and have been adjusted for the effect of egg dry mass.

We analysed both daily egg production and total number of eggs. One female laid only one egg and was removed from the analysis. The repeated measures model for daily egg production revealed a nonlinear age effect: egg production first increased, then decreased (Age: *F*_1,197_ = 15.76; *p* = 0.0001, age^2^: *F*_1,197_ = 38.36; *p* < 0.0001) (Fig 3A). The effect of family on daily egg production was significant (*F*_12,22_ = 2.81; *p* = 0.02). The flight treatment had no significant effect on egg production (*F*_1,22_ = 0.02; *p* = 0.88) and the interaction between treatment and age was also not significant (*F*_1,197_ = 1.89; *p* = 0.17). Initial body mass was added in the model as a covariate, but its effect was not significant (*F*_1,22_ = 0.01; *p* = 0.93).

The total number of eggs was not affected by the flight treatment (*F*_1,22_ = 0.01; *p* = 0.94), initial body mass (*F*_1,22_ = 0.24; *p* = 0.63) or family (*F*_13,22_ = 1.44; *p* = 0.22).The arithmetic mean number of eggs laid was 313.8 ± s.d. 161.9 in the control and 326.4±137.0 in the flight treatment. Visual inspection of the daily egg-laying data suggested that females in the flight treatment laid more eggs during the first days of adult life than control females. This was confirmed by running an ANOVA on the sum of eggs produced during the first seven days. Females in the flight treatment produced on average 87.9 ± s.d. 51.7 eggs whereas control females laid 53.7 ± 55.3 eggs. The difference in early-life fecundity was marginally significant (*F*_1,37_ = 3.98; *p* = 0.05).

### Egg dry mass and chemistry

Egg dry mass decreased with age after a moderate initial increase (age: *F*_1,120_ = 54.58; *p* < 0.0001, age^2^: *F*_1,120_ = 6.48; *p* = 0.01) (Fig 3B). The flight treatment had a significant effect on egg dry mass (*F*_1,22_ = 5.41; *p* = 0.03) but the interaction between age and treatment was not significant (*F*_1,120_ = 0.04; *p* = 0.84). There was a significant family effect on egg dry mass (*F*_13,22_ = 4.35; *p* = 0.001) (S4 Fig).

Egg protein content increased with egg dry mass (*F*_1,97_ = 5.46; *p* = 0.02), but there was no effect of age (*F*_1,97_ = 0. 08; *p* = 0.78; Fig 3C). There was a trend for higher protein content in eggs from the flight treatment, but the effect was not statistically significant (*F*_1,22_ = 3. 40; *p* = 0.08). There was no significant family effect on egg protein content (*F*_13,97_ = 1. 54; *p* = 0.12). None of the interactions between treatment and egg mass or age were significant. The Pearson correlation coefficient between protein and egg dry mass was 0.17 (*n* = 136, *p* = 0.05).

Egg triglyceride content was not affected by egg dry mass (*F*_1,99_ = 1.55; *p* = 0.22) or family (*F*_14,99_ = 0.79; *p* = 0.68. Age had no effect on triglyceride content (*F*_1,99_ = 0.51; *p* = 0.48; Fig 3D). The effect of treatment was not significant (*F*_1,36_ = 0.28; *p* = 0.60) and neither were any of the interactions between the flight treatment and covariates. The correlation between triglyceride and egg dry mass was relatively weak but significant (*r* = 0.17, *n* = 139, *p* = 0.04).

Egg glycogen content increased strongly with egg dry mass (*F*_1,78_ = 16.04; *p* = 0.0001; Fig 3E). The effect of age was not significant (*F*_1,78_ = 0.26; *p* = 0.61), and treatment had no effect on egg glycogen content (*F*_1,22_ = 0.21; *p* = 0.66). There was no family effect on glycogen (*F*_13,78_ = 0.59; *p* = 0.85). None of the interactions between treatment and the covariates were significant. Visual inspection of the data suggested that there might be a nonlinear effect of time on egg glycogen but the effect of the squared age term was not significant. The Pearson correlation coefficient between glycogen and egg dry mass was 0.50 (*n* = 117, *p* < 0.0001).

## Discussion

Increased flight significantly affected energetics and shifted the timing of reproduction but did not affect total fecundity or lifespan in the butterfly *Speyeria mormonia*. This lack of an effect on total egg production in particular contradicted our primary hypothesis that the increased energetic demand of flight would result in reduced fecundity, since fecundity is compromised when food intake is reduced in this species. The results supported our alternative hypothesis that females could compensate for increased energy demand by increasing their food intake. Indeed, both fecundity and food intake were higher in flown females compared to control females during early life. Flight metabolic rate decreased with age at a more rapid rate in flown females than in controls. This suggests that increased flight accelerated metabolic senescence, without affecting lifespan. Flight also increased resting metabolic rate, suggesting higher repair and maintenance costs due to increased energy and oxygen consumption. Furthermore, females in the flight treatment consumed more sugar water than needed to compensate for the extra energy used in flight alone, yet investment in storage did not change, as body mass did not differ between treatments. Flight therefore had effects on energetics that extended beyond direct allocation in support of flight itself, with evidence supporting increased maintenance cost. Overall, the relatively modest increase in the total energy budget had no negative effects on reproduction and survival, in spite of apparent changes in other aspects of organismal energetics and metabolism. If anything, the effects of extra flight on fitness in the wild would be positive, due to increased early reproduction, as long as food is available.

### Effects on flight metabolic rate

Both peak flight metabolic rate (PMR) and the total volume of CO_2_ emitted during the 7 min flight trial (FMR) decreased with age. However, the decrease was faster in females in the flight treatment than in control females. This pattern could be driven by the higher rate of wing wear in flown females. The reduced wing area should lead to lower drag production, which might limit metabolic output, unless it is compensated by increased wing beat frequency. However, we did not detect any significant effect of wing area on PMR or FMR, nor were there any clear trends between those variables. Even though wing wear and damage can potentially have a negative impact on flight performance and behaviour [19], there is little empirical work to demonstrate the effect in butterflies. Experimental reduction of wing size led to significantly increased wing beat frequency in a pierid butterfly, but did not affect flight activity or survival probability in the field [20]. In bumblebees, experimental wing area reduction resulted in increased mortality in the field [21] and impaired load-lifting capability [22], but not in a change in flight metabolic rate [23]. On the other hand, increased wing asymmetry was found to elevate bumblebee flight metabolic rate [24]. At this stage, the effects of wing wear and damage on insect flight energetics, particularly in Lepidoptera, are largely unknown.

Senescence or ageing refers to loss of function with advancing age. The accelerated metabolic senescence in butterflies subjected to extra flight could be due to harmful effects of increased oxidative stress. Oxidative damage has been shown to increase under metabolically demanding situations such as flight in pigeons [6], and insect flight can lead to biochemical changes that increase susceptibility to oxidative damage [25]. In the house fly, the prevention of flight decreased the accumulation rate of markers of oxidative damage, including hydrogen peroxide and protein carbonyls [5, 25]. However, the role of oxidative stress as a simple causal agent in the ageing process is unclear [26, 27]. Nevertheless, ageing insects, including Lepidoptera [28] and *Drosophila* [29, 30], show a decrease in flight capacity and other mobility-related traits. In *Drosophila*, ageing flight muscles undergo structural changes which lead to increased muscle fibre stiffness and reduced power, and mitochondria show increased levels of damage [29]. Even though the effects of ageing on insect flight metabolic rate are still poorly known, our results resemble those of Lane et al. [31], who found that flight metabolic rate decreased more rapidly with age in *D*. *melanogaster* when individuals were forced to fly, compared to control flies that were free to fly voluntarily. This suggests that, at least under certain conditions, flying insects might not enjoy the same benefits of exercise as vertebrates do.

### Resting metabolic rate

Overall, forced flight elevated RMR, which indicates increased maintenance costs of the physiological machinery, particularly given that total egg production did not change between control and flown females. Flight also increased RMR in the speckled wood butterfly *Pararge aegeria*, when male butterflies were kept either in large cages where they could fly and interact with other males or in small containers where flight was restricted [32]. In our experiment, RMR was highest in young individuals in both treatments which seems to be a common pattern among butterflies [13, 33–35] and in other insects [36]. The high RMR of young individuals likely reflects maturation processes that involve changes in muscle structure and enzymes [37, 38]. Investment in egg production also likely contributes to the high RMR in young females. *Speyeria mormonia* emerges with no mature eggs, and the first eggs are laid 3 to 4 days after emergence, after which egg production first increases, then decreases [39]. The period when the energetic cost of reproduction is at its highest therefore coincides with the period of elevated RMR.

It is noteworthy that in our experiment the difference between the two treatments increased as the individual aged: control individuals experienced a clear decrease in RMR after the first days whereas flown individuals maintained higher RMR as they aged. The high RMR of females in the flight treatment could reflect increased investment in repair and maintenance due to harmful effects of repeated flight. In fruit flies, RMR increased in ageing individuals, probably as a consequence of investment in defence mechanisms or somatic repair [40, 41]. Investment in flight can also stimulate nutrient re-allocation within the female. In a separate analysis of egg nitrogen and carbon stable isotopes, we found that flight increased the amount of carbon derived from the adult diet compared to larval-derived carbon in *S*. *mormonia* eggs [42]. The ^15^N isotope, on the other hand, was depleted in eggs from females in the flight treatment, suggesting mobilisation of stored nitrogen, as the adult diet was nitrogen-free. Nectar-feeding Lepidoptera are known to synthesise non-essential amino acids from carbon derived from the adult diet [43], and increased energy use could affect various turnover processes, thus elevating RMR.

### Investment in survival

Flight had no effect on lifespan. This is surprising as two recent studies showed that seemingly trivial amounts of flight can have strong effects on adult lifespan. Gibbs and Van Dyck [16] subjected one-day-old *Pararge aegeria* butterflies to a one-time, five-minute flight treatment. The treatment shortened lifespan by one fifth in butterflies that originated from woodlands, but had no effect in butterflies from agricultural landscapes where the demand for flight appears to be high. Saastamoinen *et al*. [17] used a similar, one-time five-minute flight treatment using the tropical butterfly *Bicyclus anynana*. Flight shortened lifespan in *B*. *anynana*, but curiously not among butterflies subjected to experimental food shortage during the larval stage. The strength of the negative effects of flight in these studies is quite remarkable when compared to our study in which females were forced to fly 3 x 4 min every day beginning the second day after eclosion. Shorter bouts of flight with rest between them might be less harmful to the butterfly than slightly longer periods of continuous flight, but we also suggest that the explanation may lie in adaptation to flight as a response to landscape configuration. *Speyeria mormonia* is a mobile species that inhabits typically continuous montane meadow landscapes with open population structure. The high mobility of *S*. *mormonia* parallels that of *P*. *aegeria* in agricultural landscapes, which suggests that adaptation to frequent flight may obviate the effects of additional flight on lifespan. Examination of other species with differing habitat structures, life styles or altitudinal conditions should be informative. For example, Marden et al. [44] showed that in the butterfly *Melitaea cinxia* adaptation to flight is manifested as individual variation in morphology and gene expression. Greater volume and elaboration of the tracheal system was connected with high flight metabolic rate and lower hypoxia-induced signalling, while individuals without these properties appeared to be less tolerant to repeated flight as they showed decreased metabolic performance and increased mitochondrial damage.

### Effects of flight on reproduction

The energetic cost of flight could lead to direct trade-offs between flight and investment in eggs. While migrating Lepidoptera rely largely on lipids to fuel flight [45], the short flight bouts of non-migratory Lepidoptera are probably primarily fuelled by carbohydrates [46, 47], and *S*. *mormonia* deposits large amounts of carbohydrate in its eggs. Here, over 1/3 of the egg dry mass consisted of glycogen, and egg glycogen content was strongly positively correlated with egg dry mass. Nevertheless, our butterflies were able to fulfil the requirements of egg production when subjected to extra flight, as food was available in abundance. In fact, early-life fecundity was higher in females in the flight treatment than in control females, although total fecundity was not affected. Furthermore, flown females laid eggs with higher dry mass than control females. This indicates strong investment in early-life reproduction and suggests that there was no trade-off between egg number and egg size.

In contrast, such a trade-off has been found e.g. in *B*. *anynana* under thermally stressful conditions [48]. Interestingly, experiments where *P*. *aegeria* females were forced to fly for 5 min either once or on three days also resulted in reduced reproductive success. These effects included lower number of eggs (but no effect on egg mass) and a negative effect of flight on offspring larval mass and development time [49]; reduced egg size [16], and decreased egg water content whereas egg carbon and nitrogen contents were unaffected [50]. This raises the question of whether the observed effects of flight in *P*. *aegeria* were mediated by actual resource shortage due to the energetic demand of flight, or whether flight at a young age, prior to the onset of reproduction, can cause a female to shift her resource investment strategy. In either case, the effects of stress are not straightforward, and conditions during larval development can interact with stress experienced at the adult stage [17, 51].

Our experiment however shows that butterflies can be surprisingly robust since none of our reproduction-related traits or lifespan was negatively affected by flight. In the field, however, increasing food intake requires an abundance of available nectar sources and when these are not available, fecundity may be compromised [52]. As we did not directly measure survival of the offspring, our choice of metrics could have missed some effects on fitness. Flight stress could also have indirect effects on fitness, such as reduced larval survival due to relaxed oviposition site choice by stressed females. Increased need for feeding also consumes time that could be used for reproduction, and may increase mortality risk.

Egg dry mass decreased with age in both treatments. Though females in the flight treatment consumed more sugar-water and had higher investment in early-life egg-production, egg dry mass inevitably decreased as females aged. Larger eggs have in some butterflies been shown to produce larger or fitter offspring [53–55]. Decreasing eggs size with age could indicate diminishing critical resources, functional senescence, or adaptation to conditions where early-life fecundity is rewarded due to high female mortality [56]. In some cases adaptive explanations for egg size dynamics have however been refuted as egg size simply appears to reflect female body size [57]. Indeed, egg size alone does not explain offspring quality, as egg composition could be more important [58, 59]. However, in our experiment, flight had no effect on the glycogen, protein or triglyceride content of eggs. Interestingly, female age had no direct effect on egg composition independent of egg dry mass. The absolute amount of glycogen allocated to eggs decreased with age but the decrease was in proportion to egg size. This differs from the case of the parasitic wasp *Eupelmus vuilletti*, in which carbohydrate, lipid and protein all decreased with age [56]. In the house fly, egg lipid content decreased with age, whereas carbohydrates, proteins and total energy content were not affected [59]. At this stage, further studies are needed to disentangle the dynamics of egg composition with age in *S*. *mormonia* and other insects with different life history strategies. We detected a family effect on egg dry mass which suggests genetic, maternal or developmental effects on egg size. In contrast, we do not know the dynamics of egg water content, even though water is a resource that is consumed during flight and is a vital component of eggs. Compared to many other insects and butterflies, the eggs of *S*. *mormonia* have a lower relative protein content [39]. Presumably the high carbohydrate and glycogen content in particular is related to the life history which is characterised by unfed first instar larvae spending the winter in diapause, relying on only maternal resources [39]. This strategy could at least partly explain why the composition of *S*. *mormonia* eggs appears to be highly conserved.

## Supporting Information

S1 Appendix Detailed methods. (PDF)

S1 Fig Effects of flight, age and family on body mass. (PDF)

S2 Fig Effects of flight, age and family on resting metabolic rate. (PDF)

S3 Fig Effects of flight, age and family on peak metabolic rate. (PDF)

S4 Fig Effects of flight, age and family on daily egg production. (PDF)

S5 Fig Effects of flight, age and family on egg dry mass. (PDF)

## References

[1] Lee I-M, Paffenbarger RS. Associations of light, moderate, and vigorous intensity physical activity with longevity: the Harvard alumni health study. Am J Epidemiol. 2000;151(3):293–9. 10670554 10.1093/oxfordjournals.aje.a010205

[2] Holloszy JO. Exercise increases average longevity of female rats despite increased food intake and no growth retardation. J Gerontol. 1993;48(3):B97–B100. . 8482812 10.1093/geronj/48.3.b97

[3] Piazza N, Gosangi B, Devilla S, Arking R, Wessells R. Exercise-training in young *Drosophila melanogaster* reduces age-related decline in mobility and cardiac performance. PLoS ONE. 2009;4(6):e5886 10.1371/journal.pone.0005886 19517023 PMC2691613

[4] Agarwal S, Sohal RS. DNA oxidative damage and life expectancy in houseflies. Proc Natl Acad Sci U S A. 1994;91(25):12332–5. 10.1073/pnas.91.25.12332 . 7991627 PMC45431

[5] Yan LJ, Sohal RS. Prevention of flight activity prolongs the life span of the housefly, *Musca domestica*, and attenuates the age-associated oxidative damage to specific mitochondrial proteins. Free Radical Biology and Medicine. 2000;29(11):1143–50. . 11121722 10.1016/s0891-5849(00)00423-8

[6] Costantini D, Dell'Ariccia G, Lipp HP. Long flights and age affect oxidative status of homing pigeons (*Columba livia*). J Exp Biol. 2008;211(3):377–81. 10.1242/jeb.012856 . 18203993

[7] Bartholomew GA, Casey TM. Oxygen-consumption of moths during rest, pre-flight warm-up, and flight in relation to body size and wing morphology. J Exp Biol. 1978;76(10):11–25. .

[8] Nudds RL, Bryant DM. The energetic cost of short flights in birds. J Exp Biol. 2000;203(10):1561–72. . 10769218 10.1242/jeb.203.10.1561

[9] van Noordwijk AJ, de Jong G. Acquisition and allocation of resources—their influence on variation in life-history tactics. Am Nat. 1986;128(1):137–42. 10.1086/284547 .

[10] Boggs CL. Understanding insect life histories and senescence through a resource allocation lens. Funct Ecol. 2009;23(1):27–37. 10.1111/j.1365-2435.2009.01527.x .

[11] Speakman JR, Mitchell SE. Caloric restriction. Mol Asp Med. 2011;32(3):159–221. 10.1016/j.mam.2011.07.001 . 21840335

[12] Boggs CL, Ross CL. The effect of adult food limitation on life history traits in *Speyeria mormonia* (Lepidoptera: Nymphalidae). Ecology. 1993;74(2):433–41. 10.2307/1939305

[13] Niitepõld K, Perez A, Boggs CL. Aging, life span, and energetics under adult dietary restriction in Lepidoptera. Physiol Biochem Zool. 2014;87(5):684–94. 10.1086/677570 25244380

[14] Guerra PA. Evaluating the life-history trade-off between dispersal capability and reproduction in wing dimorphic insects: a meta-analysis. Biological Reviews. 2011;86(4):813–35. 10.1111/j.1469-185X.2010.00172.x . 21199288

[15] Roff DA, Fairbairn DJ. Wing dimorphisms and the evolution of migratory polymorphisms among the Insecta. Am Zool. 1991;31(1):243–51. .

[16] Gibbs M, Van Dyck H. Butterfly flight activity affects reproductive performance and longevity relative to landscape structure. Oecologia. 2010;163(2):341–50. 10.1007/s00442-010-1613-5 . 20372930

[17] Saastamoinen M, Sterren Dvd, Vastenhout N, Zwaan Bas J, Brakefield Paul M. Predictive adaptive responses: Condition-dependent impact of adult nutrition and flight in the tropical butterfly *Bicyclus anynana* . The American Naturalist. 2010;176(6):686–98. 10.1086/657038 20955012

[18] Giron D, Rivero A, Mandon N, Darrouzet E, Casas J. The physiology of host feeding in parasitic wasps: implications for survival. Funct Ecol. 2002;16(6):750–7. 10.1046/j.1365-2435.2002.00679.x .

[19] Foster DJ, Cartar RV. Wing wear affects wing use and choice of floral density in foraging bumble bees. Behav Ecol. 2011;22(1):52–9. 10.1093/beheco/arq160 .

[20] Kingsolver JG. Experimental analyses of wing size, flight, and survival in the western white butterfly. Evolution. 1999;53(5):1479–90. 10.2307/2640894 . 28565569

[21] Cartar RV. Morphological senescence and longevity—an experiment relating wing wear and life-span in foraging wild bumble bees. J Anim Ecol. 1992;61(1):225–31. 10.2307/5525 .

[22] Johnson SA, Cartar RV. Wing wear, but not asymmetry in wear, affects load-lifting capability in bumble bees Bombus impatiens. Can J Zool-Rev Can Zool. 2014;92(3):179–84. 10.1139/cjz-2013-0229 .

[23] Hedenström A, Ellington CP, Wolf TJ. Wing wear, aerodynamics and flight energetics in bumblebees (*Bombus terrestris*): an experimental study. Funct Ecol. 2001;15(4):417–22.

[24] Skandalis DA, Darveau CA. Morphological and physiological idiosyncrasies lead to interindividual variation in flight metabolic rate in worker bumblebees (*Bombus impatiens*). Physiol Biochem Zool. 2012;85(6):657–70. 10.1086/665568 . 23099463

[25] Magwere T, Pamplona R, Miwa S, Martinez-Diaz P, Portero-Otin M, Brand MD, et al Flight activity, mortality rates, and lipoxidative damage in *Drosophila* . J Gerontol Ser A-Biol Sci Med Sci. 2006;61(2):136–45. . 16510857 10.1093/gerona/61.2.136

[26] Edrey YH, Salmon AB. Revisiting an age-old question regarding oxidative stress. Free Radical Biology and Medicine. 2014;71:368–78. 10.1016/j.freeradbiomed.2014.03.038 . 24704971 PMC4049226

[27] Speakman JR, Selman C. The free-radical damage theory: Accumulating evidence against a simple link of oxidative stress to ageing and lifespan. BioEssays. 2011;33(4):255–9. 10.1002/bies.201000132 . 21290398

[28] Ahman M, Karlsson B. Flight endurance in relation to adult age in the green-veined white butterfly *Pieris napi* . Ecol Entomol. 2009;34(6):783–7. 10.1111/j.1365-2311.2009.01126.x .

[29] Miller MS, Lekkas P, Braddock JM, Farman GP, Ballif BA, Irving TC, et al Aging enhances indirect flight muscle fiber performance yet decreases flight ability in *Drosophila* . Biophys J. 2008;95(5):2391–401. 10.1529/biophysj.108.130005 18515368 PMC2517049

[30] Grotewiel MS, Martin I, Bhandari P, Cook-Wiens E. Functional senescence in *Drosophila melanogaster* . Ageing Res Rev. 2005;4(3):372–97. 10.1016/j.arr.2005.04.001 . 16024299

[31] Lane SJ, Frankino WA, Elekonich MM, Roberts SP. The effects of age and lifetime flight behavior on flight capacity in *Drosophila melanogaster* . The Journal of Experimental Biology. 2014;217(9):1437–43. 10.1242/jeb.095646 24790098

[32] Vande Velde L, Van Dyck H. Lipid economy, flight activity and reproductive behaviour in the speckled wood butterfly: on the energetic cost of territory holding. Oikos. 2013;122(4):555–62. 10.1111/j.1600-0706.2012.20747.x .

[33] Niitepõld K, Hanski I. A long life in the fast lane: positive association between peak metabolic rate and lifespan in a butterfly. J Exp Biol. 2013;216(8):1388–97. 10.1242/jeb.080739 23264490

[34] Woods WA, Wood CAL, Ebersole J, Stevenson RD. Metabolic rate variation over adult lifetime in the butterfly *Vanessa cardui* (Nymphalidae: Nymphalinae): Aging, feeding, and repeatability. Physiol Biochem Zool. 2010;83(5):858–68. 10.1086/656216 . 20695812

[35] Zebe E. Über den Stoffwechsel der Lepidopteren. Z Vergl Physiol. 1954;36(3):290–317. 10.1007/bf00298218

[36] Khazaeli AA, Van Voorhies W, Curtsinger JW. Longevity and metabolism in *Drosophila melanogaster*: Genetic correlations between life span and age-specific metabolic rate in populations artificially selected for long life. Genetics. 2005;169(1):231–42. 10.1534/genetics.104.030403 15466435 PMC1448881

[37] Schippers MP, Dukas R, McClelland GB. Lifetime- and caste-specific changes in flight metabolic rate and muscle biochemistry of honeybees, *Apis mellifera* . J Comp Physiol B-Biochem Syst Environ Physiol. 2010;180(1):45–55. 10.1007/s00360-009-0386-9 . 19578858

[38] Skandalis DA, Roy C, Darveau CA. Behavioural, morphological, and metabolic maturation of newly emerged adult workers of the bumblebee, *Bombus impatiens* . J Insect Physiol. 2011;57(6):704–11. 10.1016/j.jinsphys.2011.02.001 . 21335010

[39] O'Brien DM, Boggs CL, Fogel ML. Making eggs from nectar: the role of life history and dietary carbon turnover in butterfly reproductive resource allocation. Oikos. 2004;105(2):279–91. 10.1111/j.0030-1299.2004.13012.x .

[40] Marden JH, Rogina B, Montooth KL, Helfand SL. Conditional tradeoffs between aging and organismal performance of Indy long-lived mutant flies. Proc Natl Acad Sci U S A. 2003;100(6):3369–73. 10.1073/pnas.0634985100 . 12626742 PMC152299

[41] Melvin RG, Van Voorhies WA, Ballard JWO. Working harder to stay alive: Metabolic rate increases with age in *Drosophila simulans* but does not correlate with life span. J Insect Physiol. 2007;53(12):1300–6. 10.1016/j.jinsphys.2007.07.006 . 17915248

[42] Boggs CL, Niitepõld K. Insights from stable isotopic tracers on reproductive allocation under stress. Integrative and Comparative Biology. 2014;54(5):880–9. 10.1093/icb/icu074 24920750

[43] O'Brien DM, Fogel ML, Boggs CL. Renewable and nonrenewable resources: Amino acid turnover and allocation to reproduction in lepidoptera. Proc Natl Acad Sci U S A. 2002;99(7):4413–8. 10.1073/pnas.072346699 . 11930002 PMC123662

[44] Marden JH, Fescemyer HW, Schilder RJ, Doerfler WR, Vera JC, Wheat CW. Genetic variation in HIF signaling underlies quantitative variation in physiological and life-history traits within lowland butterfly populations. Evolution. 2013;67(4):1105–15. 10.1111/evo.12004 . 23550759

[45] Dudley R, Srygley RB. Airspeed adjustment and lipid reserves in migratory Neotropical butterflies. Funct Ecol. 2008;22(2):264–70. 10.1111/j.1365-2435.2007.01364.x .

[46] O'Brien DM. Fuel use in flight and its dependence on nectar feeding in the hawkmoth *Amphion floridensis* . J Exp Biol. 1999;202(4):441–51. . 9914151 10.1242/jeb.202.4.441

[47] Haag CR, Saastamoinen M, Marden JH, Hanski I. A candidate locus for variation in dispersal rate in a butterfly metapopulation. Proc R Soc B-Biol Sci. 2005;272(1580):2449–56. 10.1098/rspb.2005.3235 . PMC1599784 16271968

[48] Steigenga MJ, Fischer K. Ovarian dynamics, egg size, and egg number in relation to temperature and mating status in a butterfly. Entomol Exp Appl. 2007;125(2):195–203. 10.1111/j.1570-7458.2007.00610.x .

[49] Gibbs M, Breuker CJ, Hesketh H, Hails RS, Van Dyck H. Maternal effects, flight versus fecundity trade-offs, and offspring immune defence in the Speckled Wood butterfly, *Pararge aegeria* . BMC Evol Biol. 2010;10:345 10.1186/1471-2148-10-345 . 21067561 PMC2993718

[50] Gibbs M, Van Dyck H, Karlsson B. Reproductive plasticity, ovarian dynamics and maternal effects in response to temperature and flight in *Pararge aegeria* . J Insect Physiol. 2010;56(9):1275–83. 10.1016/j.jinsphys.2010.04.009 . 20416319

[51] Saastamoinen M, Rantala MJ. Influence of developmental conditions on immune function and dispersal-related traits in the Glanville fritillary (*Melitaea cinxia*) butterfly. PLoS ONE. 2013;8(11):e81289 10.1371/journal.pone.0081289 . 24278412 PMC3838396

[52] Boggs CL, Inouye DW. A single climate driver has direct and indirect effects on insect population dynamics. Ecol Lett. 2012;15(5):502–8. 10.1111/j.1461-0248.2012.01766.x 22414183

[53] Fischer K, Brakefield PM, Zwaan BJ. Plasticity in butterfly egg size: Why larger offspring at lower temperatures? Ecology. 2003;84(12):3138–47. 10.1890/02-0733 .

[54] Mousseau TA, Dingle H. Maternal effects in insect life histories. Annu Rev Entomol. 1991;36:511–34. 10.1146/annurev.en.36.010191.002455 .

[55] Gibbs M, Breuker CJ, Van Dyck H. Flight during oviposition reduces maternal egg provisioning and influences offspring development in *Pararge aegeria* (L.). Physiol Entomol. 2010;35(1):29–39. 10.1111/j.1365-3032.2009.00706.x .

[56] Giron D, Casas J. Mothers reduce egg provisioning with age. Ecol Lett. 2003;6(4):273–7.

[57] Wiklund C, Karlsson B. Egg size variation in satyrid butterflies: Adaptive vs historical, "Bauplan", and mechanistic explanations. Oikos. 1984;43(3):391–400. 10.2307/3544158

[58] Fox CW, Czesak ME. Evolutionary ecology of progeny size in arthropods. Annu Rev Entomol. 2000;45:341–69. 10.1146/annurev.ento.45.1.341 . 10761581

[59] McIntyre GS, Gooding RH. Egg size, contents, and quality: maternal-age and -size effects on house fly eggs. Can J Zool-Rev Can Zool. 2000;78(9):1544–51. 10.1139/cjz-78-9-1544 .

