# Supplementary material for: Effects of Increased Flight on the Energetics and Life History of the Butterfly Speyeria mormonia
Source: PLoS One. 2015 Oct 28;10(10):e0140104. doi: 10.1371/journal.pone.0140104 (PMC4624906; doi:10.1371/journal.pone.0140104)
Supplement: S1 Appendix — (PDF) [file pone.0140104.s001.pdf]

Kristjan Niitepõld and Carol L. Boggs

Effects of increased flight on the energetics and life history of the butterfly *Speyeria mormonia*

S1 Appendix

Detailed methods

Rearing

We collected wild females in the vicinity of the Rocky Mountain Biological Laboratory, Crested Butte, Colorado (38°57'N, 106°58'W, 2900 m asl) and brought them in to the laboratory. We collected their eggs, allowed those to hatch and stored the larvae over the winter at 5°C. This mimics the conditions in the wild, where unfed 1st instar larvae spend the winter under the snow cover. We broke diapause and placed the larvae in sibling groups in organdy bags that were tied to leaves of the potted host plant *Viola soraria*. We reared the larvae in a greenhouse with a 16:8 h light:dark cycle and a 27:16°C diurnal temperature cycle. Large larvae were transferred to mesh-covered potted violets where they moved freely and had continual access to food. We searched for pupae twice a day and removed hardened pupae when they were one day old. We weighed the pupae and placed them in individual plastic cups with a mesh top. Newly-emerged adult females were mated with unrelated males in the afternoon of the day of emergence. We weighed and marked the adult butterflies on the first full day after emergence. Females were kept in cylindrical glass cages lined with wax paper (height 20 cm, diameter 15 cm). The cages contained moist paper towel and one host plant leaf inserted in a small water-filled vial.

Respirometry

We used a cylindrical, transparent 1-l respirometry chamber through which dried and CO_2 free air was pumped at the STP-corrected rate of 1.5 l min^{-1} using a Sable Systems SS-4 subsampler (Sable Systems International, Las Vegas, NV, USA) and a Sierra mass flow controller (Sierra Instruments, Monterey, CA, USA). A differential infrared CO_2 analyzer measured CO_2 concentration (Li-Cor 7000; Li-Cor, Lincoln, NE, USA). We recorded the temperature inside the respirometry chamber using a fixed NTC thermistor probe (Sable Systems). The average

chamber temperature was 31.6° C (s.d. 0.4) across all resting metabolic rate (RMR) measurements and 31.7±0.4° C across the flight metabolic rate (FMR) measurements. The chamber was covered with a dark cloth during the RMR measurement. We used a 1.5 min recording of stable CO_2 emission to represent RMR. We then removed the cloth and exposed the butterfly to light. We stimulated the butterfly to fly as continuously as possible for 7 min by shaking and tapping the chamber whenever the individual landed. At the end of the flight trial, we stopped shaking the chamber and covered it with the dark cloth. The CO_2 concentration in the chamber then fell back to the resting level.

Egg chemistry

To determine egg protein content, we crushed the eggs in 1 ml of physiological water containing 0.001% Triton X-100 (Sigma-Aldrich, St. Louis, MO, USA) and kept the samples at 4°C for one day to dissolve the proteins. We then centrifuged the sample and transferred 800 µl of the supernatant to a test tube and added 200 µl of dye reagent (Bio-Rad, Hercules, CA, USA). We prepared a bovine serum albumin (Sigma-Aldrich, St. Louis, MO, USA) standard curve in triplicate and read the absorbance at 595 nm using a spectrophotometer (SpectraMax Plus 384). We calculated the protein content in µg per egg.

To determine egg glycogen content, we crushed the eggs in 40 µl of 2% sodium sulfate and added 300 µl of (1:2) chloroform-methanol to each sample. We centrifuged the sample at 4°C for 4 min, removed the supernatant, and washed the precipitate with 400 µl of 80% methanol. We then centrifuged the sample, removed the supernatant, and added 1 ml of anthrone reagent to that supernatant. We heated the samples at 90°C for 15 min after which we filtered the samples with a 45 micron filter. We prepared a glucose standard curve in triplicate, and read the absorbance at 625 nm. We calculated the glycogen content in µg per egg.

To determine egg triglyceride content, we crushed the eggs in 150 µl of distilled water and centrifuged the samples at 4000 rpm at 4°C for 10 min. We transferred 5 µl of the supernatant of each sample to a well in a 96-well plate and added 245 µl of Infinity triglycerides stable reagent (Thermo Scientific, Waltham, MA, USA). We mixed the sample and reagent by shaking the

plate and incubated it in the dark for 5 min. We prepared a triglyceride standard curve in triplicate and read the absorbance at 500 nm. We calculated the triglyceride content in µg per egg.

Predicting sugar water intake

We compared measured sugar water intake to predicted intake under the assumption that individuals compensated for energy consumed in the flight treatments. For each individual, we calculated the amount of energy consumed during 3x4 min of flight based on the respirometry trials. We converted ml of CO_2 to Joules assuming that only carbohydrates were burned, which results in 20.9 J per ml CO_2 (Gessaman & Nagy 1988). Assuming a 85 mg per ml density and a 16.19 J per mg energy content for granulated cane sugar, 1 mg of 25% sugar-water solution contained 3.57 J of energy. We modelled the intake of the females in the control treatment as a function of age and extracted least square means of sugar water consumption for each family. To predict the intake of females in the flight treatment, we took the age-specific food intake of their siblings in the control treatment and added the amount of sugar water corresponding to the amount of energy used in flight.

Gessaman, J.A. & Nagy, K.A. (1988) Energy metabolism - errors in gas exchange conversion factors. *Physiological Zoology,* **61,** 507-513.
