# Supplementary material for: Effects of Increased Flight on the Energetics and Life History of the Butterfly Speyeria mormonia
Source: PLoS One. 2015 Oct 28;10(10):e0140104. doi: 10.1371/journal.pone.0140104 (PMC4624906; doi:10.1371/journal.pone.0140104)
Supplement: S1 Fig — (PDF) [file pone.0140104.s002.pdf]

Kristjan Niitepõld and Carol L. Boggs

Effects of increased flight on the energetics and life history of the butterfly *Speyeria mormonia*

S1 Figure

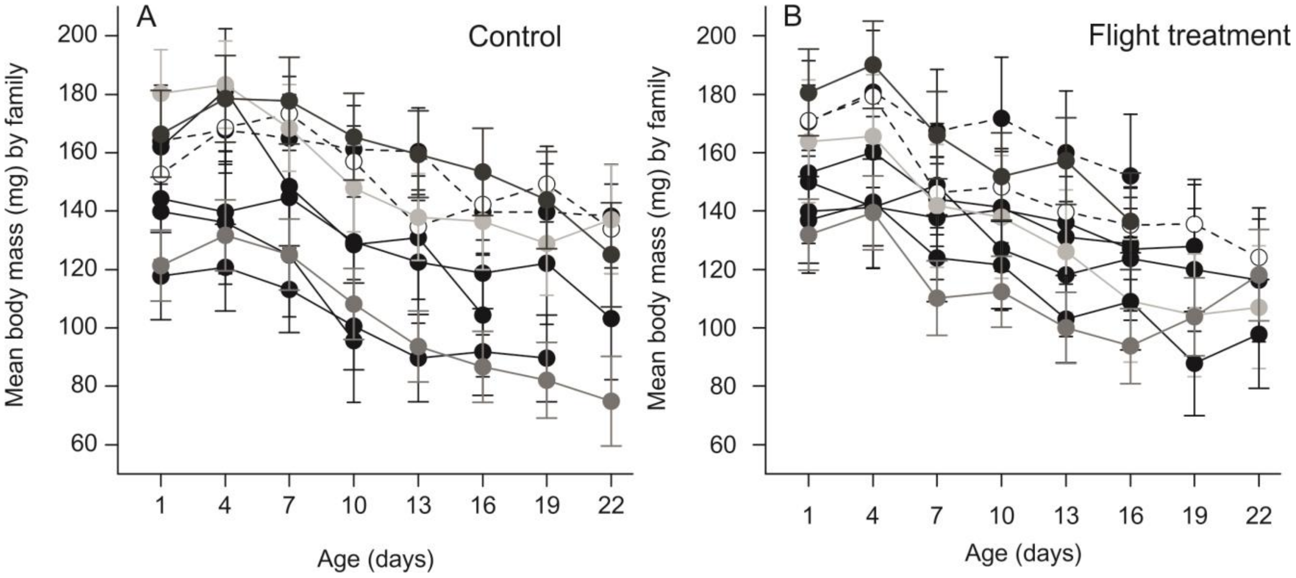

S1 Figure Least squares means of wet body mass of females across 9 families, measured every third day using the same individuals. The effect of family was significant, but the forced flight treatment had no effect on body mass. Different colors represent different families.
