# Supplementary material for: Effects of Increased Flight on the Energetics and Life History of the Butterfly Speyeria mormonia
Source: PLoS One. 2015 Oct 28;10(10):e0140104. doi: 10.1371/journal.pone.0140104 (PMC4624906; doi:10.1371/journal.pone.0140104)
Supplement: S2 Fig — (PDF) [file pone.0140104.s003.pdf]

Kristjan Niitepõld and Carol L. Boggs

Effects of increased flight on the energetics and life history of the butterfly *Speyeria mormonia*

S2 Figure

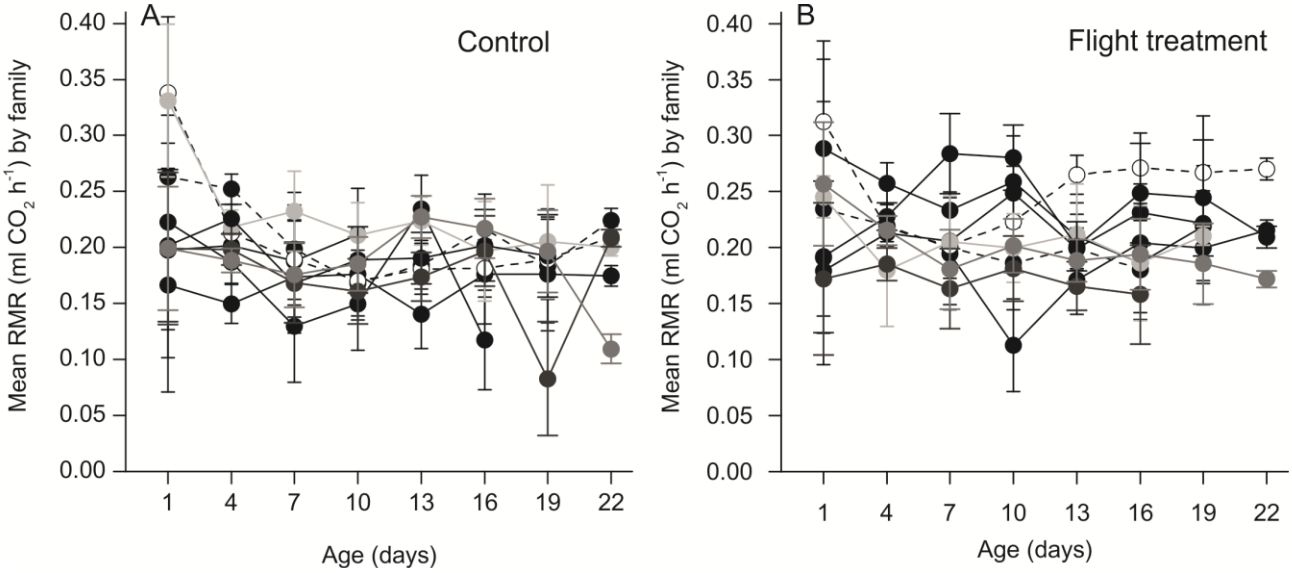

S2 Figure Least squares means of resting metabolic rate of females across 9 families, measured every third day using the same individuals. The effect of family was significant, and the forced flight treatment significantly elevated resting metabolic rate. Time since feeding and body mass had a positive effect on RMR. Different colors represent different families.
