# Supplementary material for: Effects of Increased Flight on the Energetics and Life History of the Butterfly Speyeria mormonia
Source: PLoS One. 2015 Oct 28;10(10):e0140104. doi: 10.1371/journal.pone.0140104 (PMC4624906; doi:10.1371/journal.pone.0140104)
Supplement: S4 Fig — (PDF) [file pone.0140104.s005.pdf]

Kristjan Niitepõld and Carol L. Boggs

Effects of increased flight on the energetics and life history of the butterfly *Speyeria mormonia*

S4 Figure

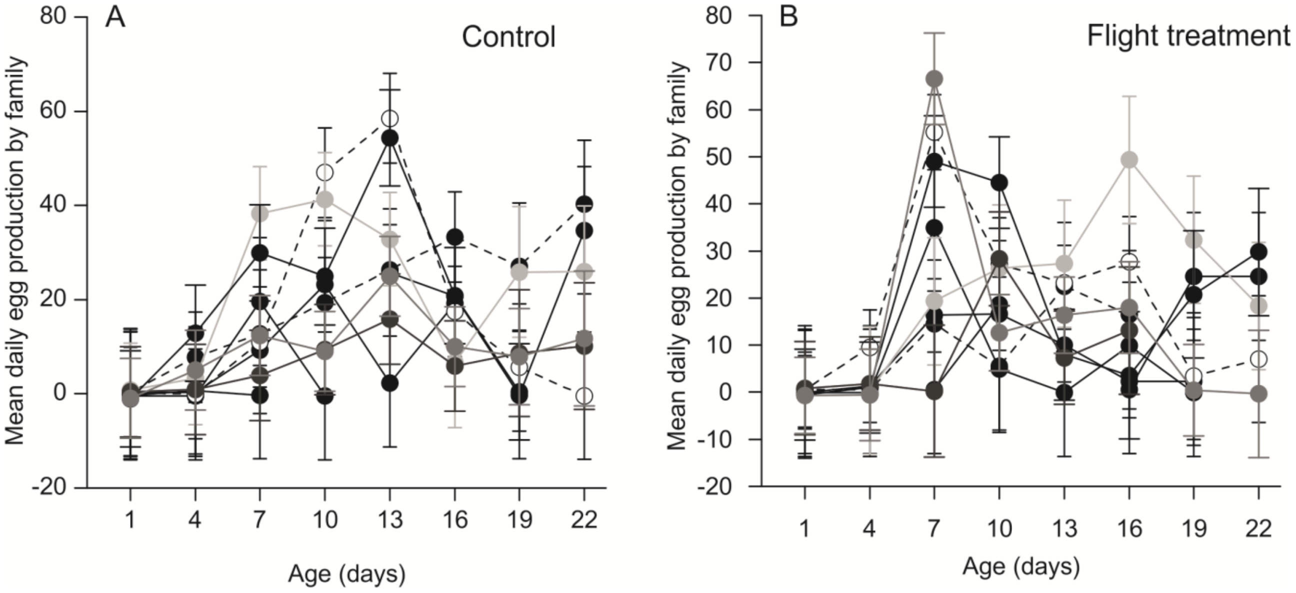

S4 Figure Least squares means of daily egg production in females across 9 families. The effect of family was significant, and there was a nonlinear effect of age, but the main effect of the flight treatment was not significant. However, a separate analysis on fecundity between ages 1-7 showed that females in the flight treatment laid more eggs in early life than control females. Different colors represent different families.
